# Integrative Analysis of Biomarkers for Cancer Stem Cells in Bladder Cancer and Their Therapeutic Potential

**DOI:** 10.3390/genes16101146

**Published:** 2025-09-27

**Authors:** Jing Wu, Wei Liu

**Affiliations:** 1 Medical School, Hubei Minzu University, Enshi 445000, China; 2 Department of Bioinformatics and Computational Biology, The University of Texas MD Anderson Cancer Center, Houston, TX 77030, USA

**Keywords:** cancer stem cell (CSC)-related genes, bladder cancer (BLCA), prognostic signature, drug sensitivity, immune infiltration, scRNA-seq

## Abstract

**Background**: Cancer stem cells (CSCs) are key drivers of tumorigenesis and metastasis. However, the precise roles of CSC-associated genes in these processes remain unclear. **Methods**: This study integrates cancer stem cell biomarkers and clinical data from The Cancer Genome Atlas (TCGA) specific to bladder cancer (BLCA). By combining differentially expressed genes (DEGs) from TCGA-BLCA samples with CSC-related biomarkers, we conducted comprehensive functional analyses and developed an 8-gene prognostic signature through Cox regression, least absolute shrinkage and selection operator (LASSO) analysis, and multivariate Cox regression. This model was validated with GEO datasets (GSE13507 and GSE32894), and the single-cell RNA seq dataset GSE222315 was subsequently analyzed to characterize the signature genes and elucidate their interactions. And a nomogram was created to stratify TCGA-BLCA patients into risk categories. The ‘oncoPredict’ algorithm based on the GDSC2 dataset assessed drug sensitivity in BLCA. **Result**: From the TCGA cohort, 665 CSC-related genes were identified, with 120 showing significant differential expression. The 8-gene signature (*ALDH1A1*, *CBX7*, *CSPG4*, *DCN*, *FASN*, *INHBB*, *MYC*, *NCAM1*) demonstrated strong predictive power for overall survival in both TCGA and GEO cohorts, as confirmed by Kaplan–Meier and ROC analyses. The nomogram, integrating age, tumor stage and risk scores, demonstrated high predictive accuracy. Additionally, the *oncoPredict* algorithm indicated varying drug sensitivities across patient groups. Based on retrospective data, we identified a novel CSC-related prognostic signature for BLCA. This finding suggests that targeting these genes could offer promising therapeutic strategies.

## 1. Introduction

Bladder cancer (BLCA) is the 10th most common cancer globally, with over 573,000 new cases reported in 2020 [1]. It is a particularly challenging disease, marked by high rates of recurrence and mortality [2]. The incidence in men is 3 to 4 times higher than in women. The 5-year survival rate for non-invasive bladder cancer is approximately 70%, but it drops sharply to less than 35% once the cancer becomes invasive. Early diagnosis and treatment are critical to improving patient outcomes. Approximately 75% of cases are non-muscle invasive bladder cancer (NMIBC), with a high recurrence rate of 50–80% [3].

The lack of reliable prognostic biomarkers in BLCA hinders identification of high-risk patients, monitoring of progression, and effective adjustment of treatment, thereby limiting improvements in patient survival and quality of life. High-throughput sequencing and molecular diagnostics offer new possibilities for personalized treatment [4]. This research focuses on the potential of tumor stem cell biomarkers, which are crucial in cancer progression and recurrence. This study aims to enhance the diagnosis and treatment of BLCA and improve patient management and survival outcomes [5].

Cancer stem cells (CSCs) represent a small subset of cancer cells with the ability to self-renew and differentiate into various tumor cell types, contributing to cancer progression, metastasis, and recurrence [6]. In 2009, bladder CSCs were first identified using markers commonly employed for isolating normal stem cells. Cell surface markers have since become the conventional method for isolating these cells [7]. Markers such as *CD44+*, *EMA−*, *67LR+*, and *BCMab1+*, found in the basal cell layer of bladder tumors, have fueled ongoing debates about the origins of bladder CSCs [8]. Recently, urothelial cancer stem cells (UroCSCs) identified in urothelial cell carcinoma (UCC) have been shown to self-renew and generate various tumor cells. They play a key role in tumor growth and survival [9]. Single-cell sequencing research by Yang et al. revealed that bladder CSCs arise not only from bladder CSCs themselves but also from bladder cancer non-stem cells (BCNSCs), indicating clonal homogeneity between the two populations. For the first time, they identified 21 critical mutation genes in bladder CSCs, including *ETS1*, *GPRC5A*, *MKL1*, *PAWR*, *PITX2*, and *RGS9BP* [10]. Moreover, the study demonstrated that co-mutations in *ARID1A*, *GPRC5A*, and *MLL2*, induced using CRISPR/Cas9 technology, significantly enhanced the self-renewal and tumor-initiating capacities of bladder CSCs [10]. Bladder CSCs are likely responsible for the high recurrence rates, tumor heterogeneity, and other complex biological behaviors observed in BLCA [8]. Despite the recognized importance of bladder CSCs, research on their relationship with bladder cancer remains limited, particularly concerning their impact on patient survival in BLCA. Therefore, this represents a critical area for future investigation.

In this study, we aimed to identify specific CSC biomarkers for predicting disease progression in BLCA. We also developed a diagnostic model focused on survival outcomes. The BCSC signature was validated using the BLCA dataset in the GEO cohort. Key genes from the set were further verified through single-cell RNA sequencing analysis. Our findings offer new insights into the diagnostic potential of CSC markers in BLCA, with implications for future research and clinical applications.

## 2. Methods and Materials

### 2.1. Data Collection and Study Design

Transcriptome profiles and clinical data for BLCA were obtained from The Cancer Genome Atlas (TCGA) database (https://portal.gdc.cancer.gov/, accessed on 31 March 2024), comprising 409 bladder cancer cases and 19 normal tissue samples. The RNA-Seq data, provided as FPKM values, were log2-transformed for downstream analysis. Additionally, the GSE13507 and GSE32894 datasets, containing RNA-Seq data and overall survival (OS) information for bladder cancer cohorts, were sourced from the Gene Expression Omnibus (GEO) database (https://www.ncbi.nlm.nih.gov/geo/, accessed on 31 March 2024). These datasets were collected retrospectively from observational cohorts and were not designed based on the intention-to-treat (ITT) principle. Single-cell RNA sequencing data from GSE222315 were also retrieved from the GEO database [11]. Data normalization, integration, dimensionality reduction, and clustering were performed following the guidelines in the Seurat manual [12].

A total of 710 biomarkers of cancer stem cells (BCSCs) were identified from the Cancer Stem Cell Database (BCSCdb) (http://dibresources.jcbose.ac.in/ssaha4/bcscdb, accessed on 31 March 2024), which aggregates experimentally validated CSC-related biomarkers from both low- and high-throughput studies on human cell lines [13]. From these, highly reliable genes were selected for detailed analysis, as listed in Table S1.

This study aimed to identify a gene signature for bladder CSC biomarkers using the TCGA-BLCA dataset. The research was carried out in two main phases. First, expression data from TCGA-BLCA were used to develop a prognostic model targeting CSCs. Next, BLCA patients in the GEO validation cohorts were stratified into different risk categories using this model. A visual summary of the methodology is shown in Figure S1.

### 2.2. Identification of Differentially Expressed BCSCs (DE-BCSCs) and Functional Enrichment Analysis

Using the TCGA-BLCA dataset, we focused on cancer stem cell (CSC)-related genes for our study. The ‘*limma*’ package in R [14] was used to identify significantly differentially expressed BCSCs, applying a threshold of |log2 fold change| > 1 and an adjusted *p*-value < 0.05. Table S4 lists 120 DE-BCSC genes.

To explore the biological significance of these DE-BCSCs, we conducted Gene Ontology (GO) [15] and Kyoto Encyclopedia of Genes and Genomes (KEGG) pathway enrichment analyses [16], using the ‘clusterProfiler’ package in R [17]. The key enrichment terms identified are highlighted in this study. Additionally, unsupervised clustering analysis was carried out using the ‘*ConsensusClusterPlus*’ package in R [18] to classify samples from both the GEO and TCGA cohorts. Using 1000 iterations, this approach determined the optimal number of clusters, ensuring robustness of our classifications [19]. All data were processed using R version 4.3.0.

### 2.3. Construction of BCSCs Prognostic Signature and Risk Model

Univariate Cox regression analyses were employed to explore the association between DE-BCSCs and OS in the TCGA-BLCA dataset. Genes with a *p*-value < 0.05 were selected for further analysis. To identify the most relevant prognostic genes, we applied the least absolute shrinkage and selection operator (LASSO) method, followed by multivariate Cox regression. The risk score for each patient was calculated using the formula: Risk score = ∑(Coef_j × X_j), where Coef_j is the coefficient from the multivariate Cox regression, and X_j represents the mRNA expression levels of each gene.

Patients were stratified into high- and low-risk groups based on their calculated risk scores, and the performance of the BCSC prognostic signature was assessed using Kaplan–Meier survival analysis. To validate the predictive power of this signature, external validation was conducted using the GSE13507 and GSE32894 cohorts.

### 2.4. Development of a Nomogram Model

We conducted univariate and multivariate Cox regression analyses to evaluate the prognostic value of BCSC risk scores. Additionally, related clinical parameters were assessed for their ability to predict OS in BLCA patients. The predictive accuracy of risk scores and clinical parameters was evaluated using receiver operating characteristic (ROC) curve analysis. A prognostic nomogram was then developed to estimate 1-, 3-, and 5-year OS rates for BLCA patients in the TCGA cohort, using the “*timeROC*” v0.4 R package [20]. We performed internal validation of this model using bootstrap resampling and presented the results in a calibration plot to visually assess its accuracy.

### 2.5. Gene Set Enrichment Analysis (GSEA) for Risk Groups in BLCA

To further investigate the molecular mechanisms underlying the prognostic power of the BCSC signature in BLCA, we performed Gene Set Enrichment Analysis (GSEA) using the “*clusterProfiler*” v4.16.0 and “*org.Hs.eg.db*” v3.12.0 packages in R [21]. This study highlights the top five most significantly enriched terms from the KEGG and Hallmark pathways for both high- and low-risk groups.

### 2.6. Immune Infiltration Analysis and Gene Mutation Information

The ESTIMATE algorithm (Estimation of Stromal and Immune cells in Malignant Tumor tissues using Expression data) was applied to calculate the immune score, stromal score, ESTIMATE score, and tumor purity for BLCA samples [22]. Additionally, the “*CIBERSORT*” v1. 0 R package was used to quantify the relative abundance of various immune cell types [23]. We also evaluated immune checkpoint gene expression as potential indicators of immunotherapy response [24]. The abundance of immune and stromal cell populations in the tumor microenvironment was estimated using TIMER 2.0 (http://timer.cistrome.org/ (accessed on 31 August 2024) [19]). Mutation data for BLCA were obtained from the TCGA database, and tumor mutation burden (TMB) was calculated using the “*maftools*” R package v2.24.0 [25]. Additionally, the top 20 genes with the highest mutation frequencies were visualized for both high-risk and low-risk groups.

### 2.7. Single Cell RNA-Seq (scRNA) Analysis for GSE222315

Original count matrices for BLCA were downloaded from GSE222315, filtered to keep cells with 200–6000 detected genes and <20% mitochondrial transcripts and genes detected in ≥3 cells. Counts were normalized to 10,000 transcripts per cell and log_10_-transformed. Then, the top 3 000 highly variable genes were used for PCA (50 components), k-nearest-neighbor graph construction (k = 15), leiden clustering (resolution = 0.5) and UMAP visualization in *Scanpy* 1.9.5 [26]. Cell identities (B, T, NK, myeloid, mast, epithelial, endothelial and fibroblast) were assigned by canonical markers [27]. Feature and dot plots were specifically generated for the eight prognostic genes (*ALDH1A1*, *CBX7*, *CSPG4*, *DCN*, *FASN*, *INHBB*, *MYC*, *NCAM1*). Ligand–receptor interactions were inferred using *LIANA* framework [28] and *CellPhoneDB* v2.1.7 [29] (1000 permutations; Benjamini–Hochberg-adjusted *p* < 0.05) and visualized as bubble plots.

### 2.8. Sensitivity of Antitumor Drugs

To evaluate chemotherapy sensitivity in the TCGA-BLCA cohort, we calculated and compared the half-maximal inhibitory concentration (IC50) values of various antitumor drugs between the two risk groups with the R package “*oncoPredict*” [30]. This tool estimates drug sensitivity by leveraging the Genomics of Drug Sensitivity in Cancer (GDSC) database.

### 2.9. Statistical Analysis

Continuous variables between two groups were compared using the Student *t*-test or Mann–Whitney U test, depending on data distribution. Survival differences were assessed using the log-rank test. Hazard ratios (HRs) and 95% confidence intervals (CIs) were estimated by univariate and multivariate Cox regression analyses. A *p* value < 0.05 was considered statistically significant unless otherwise stated.

## 3. Results

### 3.1. Expression of BCSCs in the TCGA-BLCA Cohort

**Figure 1:**
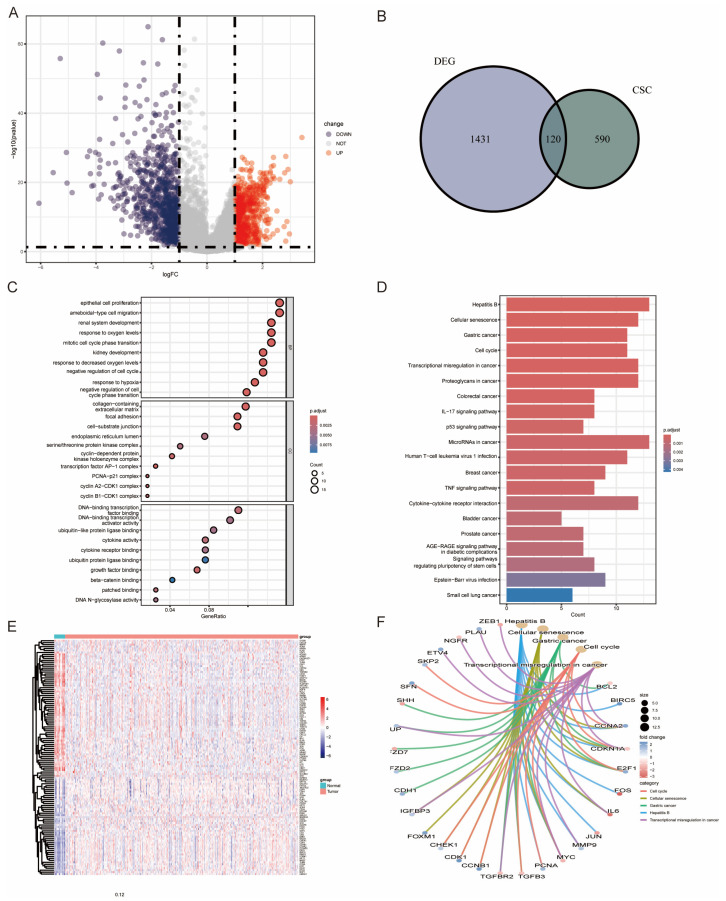
Identification of CSC-Related Genes in the TCGA-BLCA Cohort. (**A**) Differential gene expression between tumor and normal tissues. (**B**) Identification of 120 CSC-associated genes overlapping with DEGs in BLCA. (**C**–**F**) Functional enrichment analyses of these genes: GO terms (**C**), KEGG pathways (**D**), heatmap (**E**), and top five KEGG pathways (**F**).

We identified 1154 differentially expressed genes (DEGs) by comparing 402 BLCA samples with 19 normal samples. Among these, 120 genes overlapped with BCSC-related genes (Figure 1A). Of these, 65 genes were downregulated and 55 were upregulated (Figure 1B,E and Figure S2A and Table S4). To further understand the biological significance of the 120 DEGs, we performed a functional enrichment analysis (Figure 1C,D). GO analysis revealed that these genes are primarily involved in processes such as ameboid-type cell migration, response to hypoxia, cell cycle progression, and renal system development (Figure 1C). Similarly, KEGG analysis revealed significant enrichment in pathways associated with the cell cycle and various cancers, including gastric and colorectal cancers, as well as the IL-17 signaling and P53 signaling pathways (Figure 1D). The top five enriched KEGG pathways, highlighting their critical roles in BLCA pathogenesis, are shown in Figure 1F. Among them, cell cycle, cellular senescence, and transcriptional mis-regulation in cancer stand out as especially prominent.

### 3.2. Development and Validation of a CSC-Based Signature in BLCA Cohort

**Figure 2:**
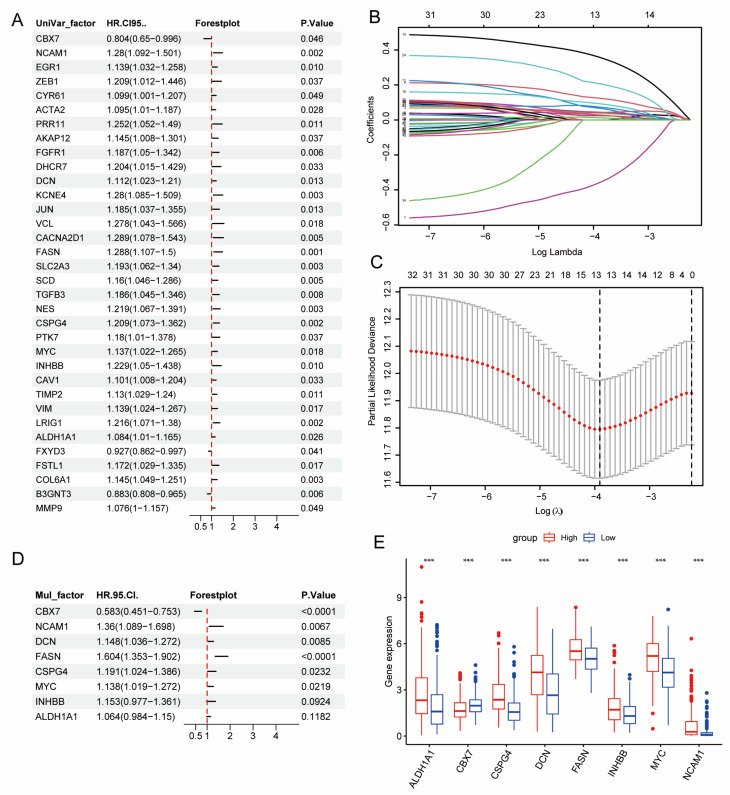
Development of the DE-BCSC Model. (**A**) Univariable Cox regression analysis identified 34 of the 120 DE-BCSC genes as significant prognostic markers for overall survival (OS) in the TCGA-BLCA cohort. (**B**,**C**) LASSO regression analysis further refined these findings, identifying 13 key genes. (**B**) Each colored line represents the coefficient path of a gene as the regularization parameter (log λ) changes, with only a subset retaining non-zero coefficients. (**C**) The red dots indicate the mean partial likelihood deviance for each log λ value, while the gray error bars represent the corresponding standard errors. (**D**) Random Forest analysis narrowed this down to 8 DE-CSCs, which were identified as critical prognostic indicators. (**E**) Expression levels of these 8 diagnostic genes were compared between high-risk and low-risk groups within the TCGA-BLCA dataset. Significance levels are indicated as * *p* < 0.05, ** *p* < 0.01, and *** *p* < 0.001.

We developed a DE-BCSC-based prognostic signature to evaluate its impact on BLCA patient outcomes. Univariate Cox regression analysis of 120 DE-BCSCs identified 34 genes significantly associated with overall survival in BLCA patients. LASSO analysis refined the set to 13 genes, and multivariate Cox regression further narrowed it to eight key genes (Figure 2A–D).

The prognostic index for each patient was derived using the formula: Risk score = (−0.54 × *CBX7* expression) + (0.307 × *NCAM1* expression) + (0.137 × *DCN* expression) + (0.472 × *FASN* expression) + (0.175 × *CSPG4* expression) + (0.13 × MYC expression) + (0.142 × *INHBB* expression) + (0.062 × *ALDH1A1* expression).

The risk score for each patient was calculated from gene expression levels, and patients in both TCGA and GEO cohorts (GSE13507 and GSE32894) were stratified into high- and low-risk groups using the median risk score. The expression profiles of the eight prognostic genes between these groups are illustrated in boxplots (Figure 2E and Figure S2B,C). Higher expression of seven genes (*ALDH1A1*, *CSPG4*, *DCN*, *FASN*, *INHBB*, *MYC*, and *NCAM1*) correlated with worse survival in the high-risk group. In contrast, CBX7 expression was higher in the low-risk group, which was associated with better prognosis. Pathway analysis for the two subgroups was detailed in Table S2.

### 3.3. Clinical Relevance of DE-BCSCs

**Figure 3:**
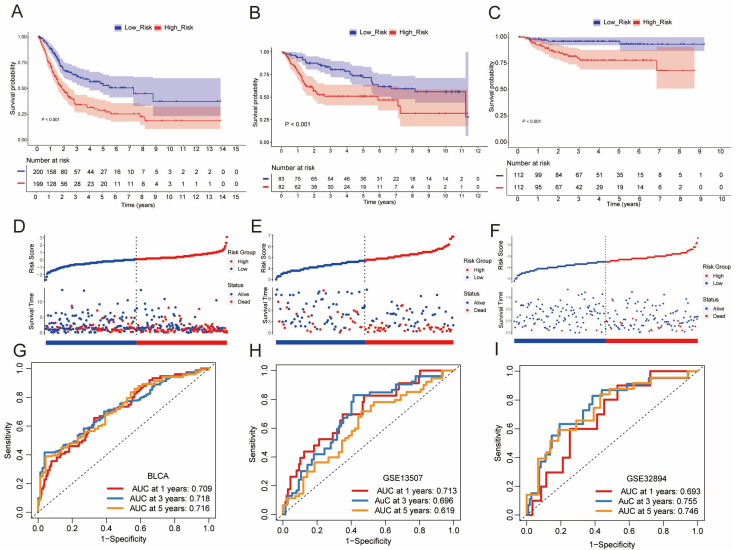
The CSC Model Serves as a Consistent Prognostic Predictor Across TCGA and GEO Cohorts. (**A**–**C**) Kaplan–Meier survival curves show that high-risk patients have significantly poorer overall survival in the TCGA-BLCA (**A**), GSE13507 (**B**), and GSE32894 (**C**) cohorts. (**D**–**F**) Risk score curves and scatter plots distinguish high-risk (red) and low-risk (blue) patients in the BLCA (**D**), GSE13507 (**E**), and GSE32894 (**F**) cohorts. (**G**–**I**) Time-dependent ROC curves illustrate the predictive accuracy of the model for overall survival in the TCGA (**G**), GSE13507 (**H**), and GSE32894 (**I**) cohorts.

Kaplan–Meier analysis assessed the relationship between the BCSC signature and OS in BLCA. The survival curves showed that patients in the high-risk group had significantly worse overall survival compared to those in the low-risk group (Figure 3A). To validate the prognostic capability of the BCSC signature, risk scores were calculated for patients in the GSE13507 and GSE32894 cohorts (Figure 3B,C). Risk score curves and scatter plots further supported the signature’s effectiveness in distinguishing between risk groups (Figure 3D–F). Time-dependent ROC curves demonstrated the CSC model’s accuracy, with stable AUC values confirming its reliability in predicting survival outcomes at different time points (Figure 3G–I).

### 3.4. Development of a Nomogram

**Figure 4:**
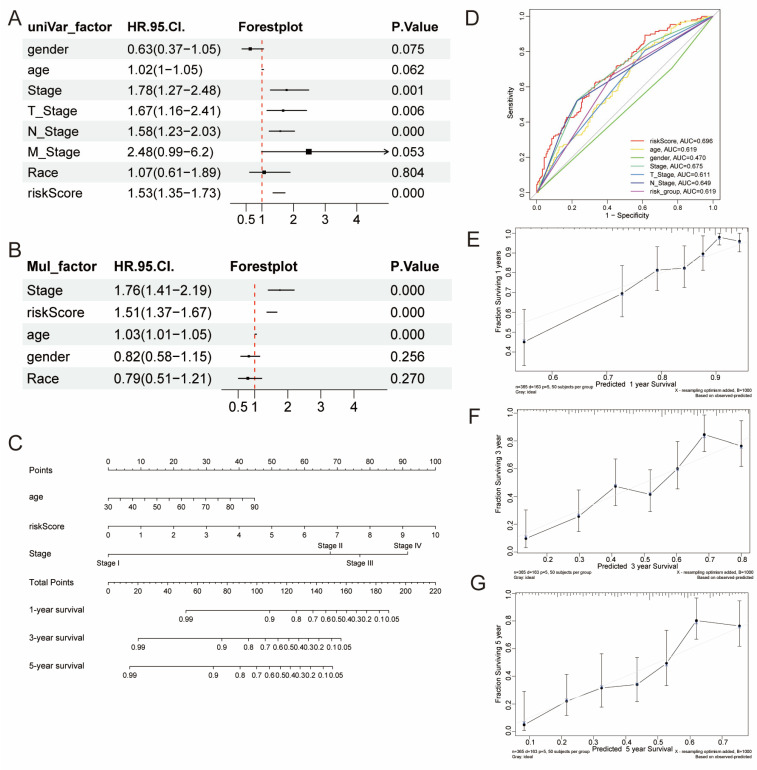
Subgroup Analyses and Development of the Prognostic Nomogram. (**A**) Univariate and (**B**) multivariate Cox regression analyses were performed on the BLCA patient cohort. (**C**) A prognostic nomogram was constructed based on the results of the multivariate Cox model in TCGA-BLCA. (**D**) The ROC curve assessed the performance of the risk score in comparison with relevant clinical factors. (**E**–**G**) Calibration curves demonstrated strong agreement between the nomogram’s predicted and actual overall survival rates at 1 year (**E**), 3 years (**F**), and 5 years (**G**) in the BLCA cohort. For a given patient, find their value on each variable axis: age, risk Score, and Stage. The black line with error bars shows the predicted versus observed survival, while the gray diagonal line represents the ideal reference line. For each variable, draw a line upward to the ‘Points’ axis to determine its score. Next, sum all scores and locate the total on the ‘Total Points’ axis. Draw a line down from the total points to the ‘1-, 3-, or 5-year Survival’ axis, and read the predicted survival probability.

To further validate clinical variables, we evaluated factors such as age, gender, cancer stage, T stage, N stage, M stage, race, and risk score using univariate and multivariate Cox regression analyses. Univariate analyses identified cancer stage, T stage, N stage, and risk score as significantly associated with OS (*p* < 0.05) (Figure 4A). The multivariate analysis further confirmed that age, cancer stage, and risk score were independent predictors of OS (*p* < 0.05) (Figure 4B). Notably, the risk score showed superior predictive accuracy compared to other clinical variables, with an AUC of 0.696 in the ROC curve (Figure 4D). A nomogram was then constructed using age, stage and risk score to predict 1-, 3-, and 5-year OS (Figure 4C). Calibration curves confirmed the nomogram’s accuracy, showing strong agreement between predicted and actual OS outcomes (Figure 4E–G).

### 3.5. Gene Set Enrichment Analysis (GSEA)

**Figure 5:**
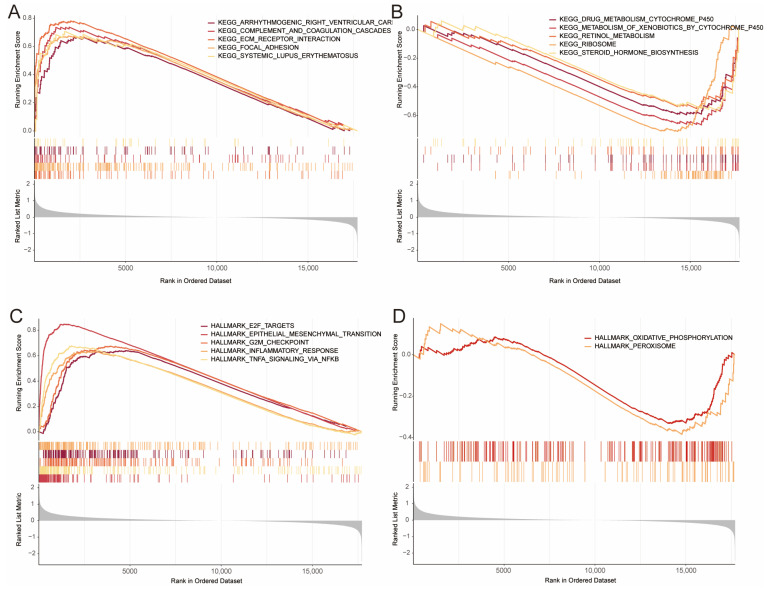
GSEA of High- and Low-Risk Group Samples. (**A**,**B**) Gene Set Enrichment Analysis (GSEA) highlights enriched gene sets from the C2 collection, showing the top five KEGG pathways for up-regulated (**A**) and down-regulated (**B**) genes. (**C**,**D**) Analysis of HALLMARK gene sets reveals the top five enriched pathways for up-regulated genes (**C**) and the top two pathways for down-regulated genes (**D**).

GSEA was conducted to investigate the molecular mechanisms underlying the DE-BCSCs signature in the TCGA cohort. In the high-risk group, the top five enriched KEGG pathways included ECM receptor interaction, focal adhesion, complement and coagulation cascades, ARVC, and systemic lupus erythematosus. In contrast, the low-risk group showed prominent enrichment of pathways related to steroid hormone biosynthesis, retinol metabolism, and cytochrome P450-mediated drug metabolism (Figure 5A,B and Table S3).

In the high-risk group, hallmark pathways such as epithelial–mesenchymal transition (EMT), TNF-α/NF-κB signaling, and the G2M checkpoint were significantly enriched. While the low-risk group showed enrichment in oxidative phosphorylation and peroxisome pathways (Figure 5C,D). GO and KEGG analyses further highlighted significant enrichment in processes such as skin development, ECM-receptor interaction, and PI3K-Akt signaling (Supplementary Figure S3A,B).

### 3.6. Association of Risk Score with Clinical Factors

**Figure 6:**
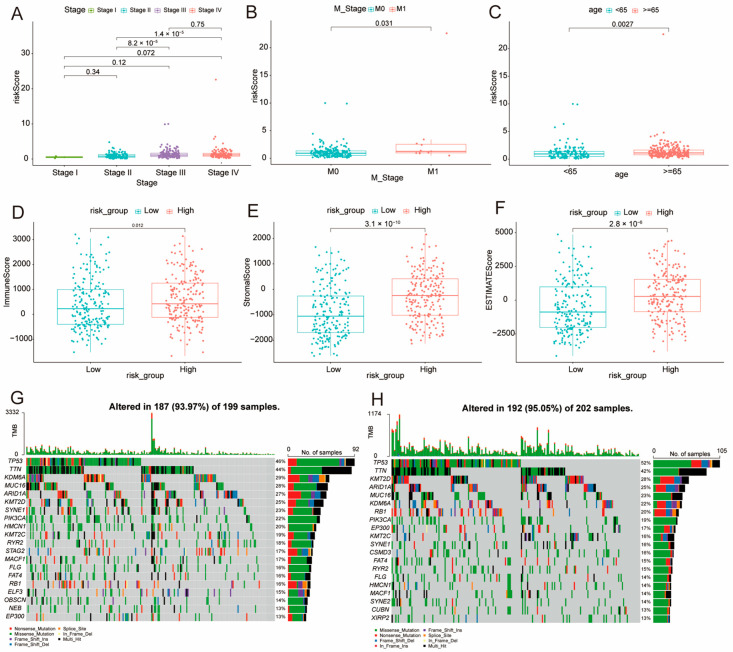
Risk Score and Clinical Correlation Analysis. (**A**–**C**) Comparative analysis of risk scores across cancer stages, M stages, and age groups, respectively. (**D**–**F**) Evaluation of immune-related scores between high- and low-risk groups, highlighting variations in immune profiles within these classifications. (**G**,**H**) Oncoprint visualizations for the low-risk group (**G**) and high-risk group (**H**), illustrating the genetic landscape and mutation patterns in each group.

Our analysis showed that higher risk scores were associated with more advanced cancer stages. Significant differences were observed between stage II and stage III (*p* = 8.2 × 10^−5^), as well as between stage II and stage IV (*p* = 1.4 × 10^−5^) (Figure 6A). Additionally, significant differences in risk scores were found between metastatic stages (M1 vs. M0, *p* = 0.031) and across age groups (*p* = 0.0027) (Figure 6B,C). Using the ESTIMATE algorithm, we found marked differences in ESTIMATE, immune, and stromal scores between the low- and high-risk groups (*p* < 0.001) (Figure 6D–F). To compare the mutational landscapes between the two risk groups, we depicted the top 20 most frequently mutated genes for each group. Further genomic analysis revealed higher mutation rates in the high-risk group (95.05%) compared to the low-risk group (93.97%). Notably, TP53 mutations increased from 46% in the low-risk group to 52% in the high-risk group, while KDM6A mutations decreased from 29% to 20% (Figure 6G,H).

### 3.7. Immune Infiltration in Low- and High-Risk Groups

**Figure 7:**
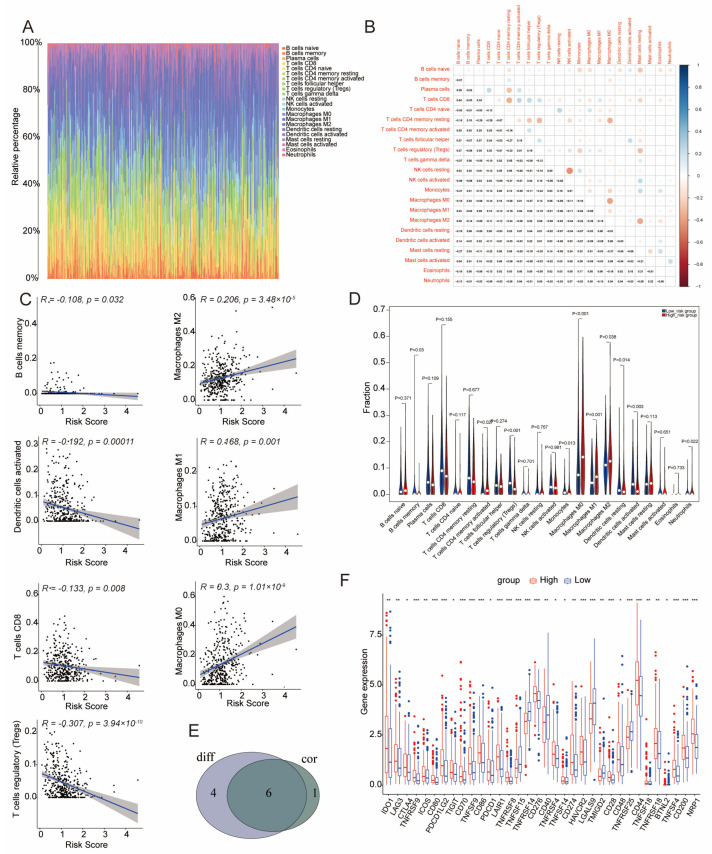
Immune-Related Analysis. (**A**) Comprehensive assessment of immune cell populations in tumor samples. (**B**) Correlation analysis between different immune cell types. (**C**) Regression analysis between immune cell populations and the risk score; black dots represent individual samples, the blue line indicates the fitted regression line, and the gray area shows the 95% confidence interval. (**D**) Comparison of immune cell distributions between high- and low-risk groups. (**E**) Overlap analysis of immune cells that are both differentially expressed and correlated with risk. (**F**) Variations in immune-related genomic features between high- and low-risk groups.

A heatmap of immune cell infiltration, analyzed via the CIBERSORT algorithm, is shown in Figure 7A. Significant correlations were found between CD8 T cells and CD4 memory resting T cells, between M2 macrophages and CD4 memory resting T cells, and between M2 and M0 macrophages. Strong correlations were also observed between activated and resting NK cells (Figure 7B). We used linear regression analyses to quantify the association between the risk score and immune infiltration. We observed statistically significant but weak positive or negative correlations between the risk score and seven immune cell types (*p* < 0.05, Figure 7C). Higher risk scores were associated with increased infiltration of immune cells such as CD4 memory activated T cells, M0, M1, and M2 macrophages, and neutrophils (*p* < 0.05, Figure 7D). Further analysis revealed substantial overlap among different immune cell types (Figure 7E).

Expressions of multiple immune checkpoints, including *IDO1*, *LAG3*, *CTLA4*, *TNFRSF9*, *ICOS*, and others, were significantly higher in the high-risk group. Conversely, checkpoints such as *TNFSF15*, *TNFSF14*, *CD40*, *LGALS9*, *TMIGD2*, *TNFRSF25*, and *BTNL2* were expressed at lower levels (Figure 7F). These findings underscore the critical role of immune profiling in evaluating risk and predicting outcomes in BLCA.

### 3.8. ScRNA-Seq Analysis of the Signature Genes

**Figure 8:**
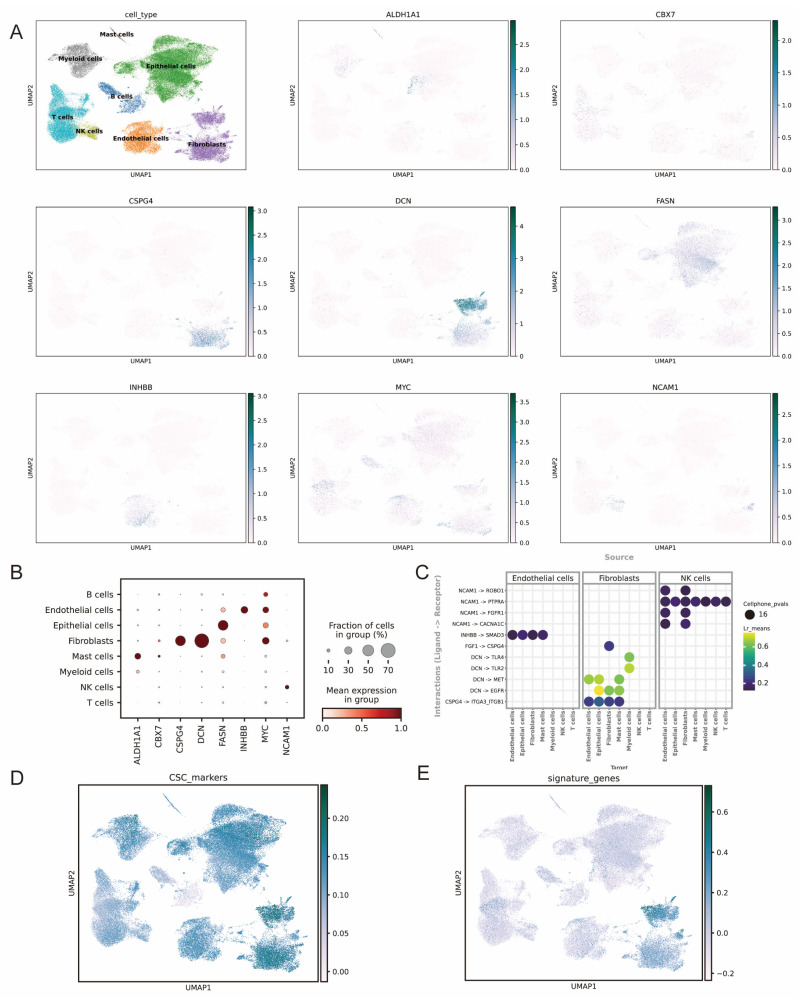
Tumor microenvironment cell states and hub genes’ expression (scRNA-seq). (**A**) The UMAP shows 8 cell populations along with feature plots for 8 hub genes, including *ALDH1A1*, *CBX7*, *CSPG4*, *DCN*, *FASN*, *INHBB*, *MYC*, and *NCAM1*. (**B**) The expression of the signature genes among the defined cell types. (**C**) A ligand–receptor–based cell–cell communication network shows interactions linked to the hub genes. (**D**) The UMAP plot shows the distribution of CSC markers in cell clusters. (**E**) UMAP plot displays 8 signature genes across all clusters.

We interrogated the scRNA-seq dataset GSE222315 to delineate the expression and signaling roles of eight prognostic genes in BLCA. Unsupervised clustering identified eight tumor microenvironmental populations, including B cells, T cells, NK cells, myeloid cells, mast cells, epithelial cells, endothelial cells, and fibroblasts (Figure 8A). Feature plots revealed pronounced cell-type specificity: *DCN* was almost exclusive to fibroblasts and showed the highest average abundance; *NCAM1* dominated NK and endothelial compartments; *FASN* was enriched in epithelial cells, and CSPG4 in fibroblast subsets. In contrast, *ALDH1A1*, *CBX7*, *INHBB* and *MYC* were detected in <30% of any lineage (Figure 8A,B). We comprehensively mapped the expression patterns of BCSC genes across all identified cell populations (Figure 8D). Notably, fibroblast populations predominantly and actively expressed the eight prognostic signature genes (Figure 8E). This expression pattern aligns with our bulk RNA-seq immune infiltration analysis. We used multiple deconvolution algorithms in TIMER 2.0 to analyze the data. Our results showed a strong and significant association between these eight signature genes and Cancer-Associated Fibroblast (CAF) infiltration levels (see Table S5 for detailed results). The agreement between single-cell data and bulk tissue analysis strongly highlights the pivotal role of CAFs in the biology of our identified gene signature.

CellPhoneDB ligand–receptor mapping (adjusted *p* < 0.05) uncovered 16 significant interactions for hub genes, partitioned into three source-cell modules (Figure 8C). Fibroblast-derived *DCN* predominated. It signaled to myeloid (TLR2/4) and epithelial (EGFR/MET) cells (LR_mean ≤ 0.70), and as a result, coupled immune activation to epithelial proliferation. A single high-confidence *CSPG4* → *ITGA3/ITGB1* interaction conveyed fibroblast-to-epithelial adhesion. Endothelial cells engaged in an autocrine *INHBB* → *SMAD3* loop, while low-amplitude *NCAM1* contacts linked endothelial and NK cells. No extracellular pairs were detected for *ALDH1A1*, *CBX7*, *FASN* or *MYC*, indicating primarily intracellular roles.

### 3.9. Drug Sensitivity Analysis

**Figure 9:**
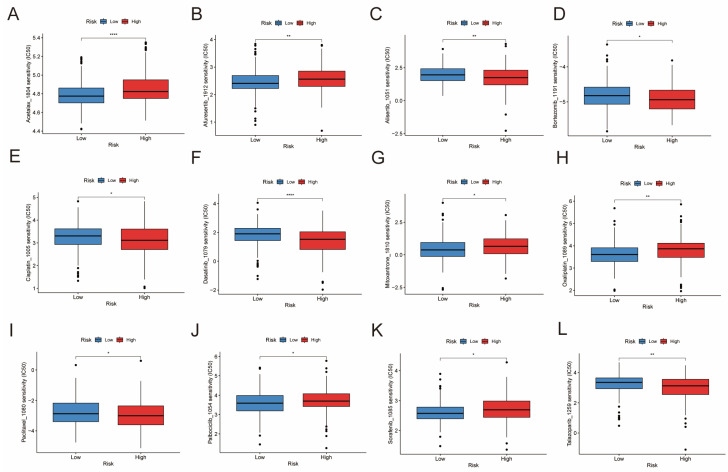
Comparison of chemotherapy drug sensitivity between low- and high-risk subgroups. The oncoPredict algorithm predicted the IC50 values of Acetalax (**A**), Afuresertib (**B**), Alisertib (**C**), Bortezomib (**D**), Cisplatin (**E**), Dasatinib (**F**), Mitoxantrone (**G**), Oxaliplatin (**H**), Paclitaxel (**I**), Palbociclib (**J**), Sorafenib (**K**), and Talazoparib (**L**) in the two subgroups. Significance levels are indicated as * *p* < 0.05, ** *p* < 0.01, *** *p* < 0.001, and **** *p* < 0.0001.

To evaluate the predictive utility of the risk model, we further investigated the differences in IC50 levels of chemotherapeutic drugs across risk subgroups. This analysis used data from the GDSC2 database, which includes 198 drugs in total. The results indicate that patients in the low-risk group have lower IC50 values for several anti-cancer drugs, including Acetalax, Afuresertib, Mitoxantrone, Oxaliplatin, Palbociclib, and Sorafenib. Conversely, the high-risk group showed lower IC50 values for drugs such as alisertib, bortezomib, cisplatin, dasatinib, paclitaxel, and talazoparib (Figure 9A–L). These results highlight distinct drug sensitivity profiles between the two risk groups. These findings indicate that the DE-BCSC signature may serve as a predictive biomarker for drug sensitivity and therapy selection.

## 4. Discussion

In our study, we developed and validated a prognostic model based on eight BCSC-related genes to predict overall survival in BLCA patients. We integrated the identified genes into a prognostic BCSC model that stratified patients into two distinct risk groups in the TCGA-BLCA and GEO datasets. These groups demonstrated significant differences in clinical features, survival outcomes, biological functions, and immunological profiles. Patients in the high-risk group showed poorer survival, emphasizing the prognostic value of the BCSC gene signature and offering new insights into CSCs’ role in BLCA progression and prognosis.

In recent years, many studies have shown that BCSCs, a specialized gene set in cancer cells, are crucial in driving multiple cancer progression and metastasis. For example, the expression of stem cell markers such as *SOX2*, *IGF1R*, *SOX4*, *ALDH1*, and *CD44* affects the likelihood of recurrence and metastasis in bladder cancer [31,32]. Similarly, research by Yao Kang et al. has demonstrated that an mRNA expression-based stemness index (mRNAsi) can predict tumorigenesis, bone metastasis, and patient prognosis in bladder cancer [33]. Our results align with these findings, further highlighting the importance of CSCs in tumor initiation, metastasis, and therapy.

Most key genes in our model contribute to a higher risk score. For example, *ALDH1A1*, *CSPG4*, *DCN*, *FASN*, *INHBB*, *MYC*, and *NCAM1* showed significantly lower expression in the low-risk group than in the high-risk group. This finding supports previous research. ALDH1A1, a recognized diagnostic target overexpressed in bladder cancer, and its downstream target TUBB3 can be degraded to reduce invasion and proliferation, providing new avenues for diagnosis and therapy in advanced disease [34,35]. Similarly, CSPG4 is a key gene associated with EMT, energy metabolism, prognosis, and immune infiltration in bladder cancer, with high expression correlating with poorer prognosis [36]. Additionally, CSPG4’s expression and mutations may influence immune cell behavior, further impacting BLCA prognosis [37]. *DCN* (decorin) activates the *MAPK* pathway by binding with epidermal growth factor. This activation leads to the expression of downstream p21 genes, influencing tumor differentiation, invasion depth, hyperplasia, metastasis, and prognostic significance [38,39]. Suppressing *ΔDCN* expression leads to a decrease in BCSC stemness [40]. Upregulation of FASN has been observed in multiple cancers, including BLCA, and is associated with poor prognosis [41]. *FASN* independently predicts prognosis and regulates bladder cancer cell proliferation and metastasis via the Wnt/β-catenin pathway [42]. *INHBB*, a member of the TGF-β family, influences DNA synthesis in various tumors and promotes proliferation and invasiveness in glioblastoma [43]. In this study, we found that high *INHBB* expression correlates with poor prognosis in bladder cancer patients, a finding consistent with previous reports [44]. *MYC* is a transcription factor known to be involved in tumor development and modulation of the tumor microenvironment. Knockdown of *MYC* in BLCA cell lines has been reported to inhibit proliferation and metastasis [45]. Wang Y et al., reported that DNA polymerase *POLD1* promotes BLCA proliferation and metastasis through MYC [46]. *NCAM1*, a cell adhesion protein, may reduce cell invasion and potentially contribute to bladder cancer recurrence [28]. It acts as a suppressor of cell proliferation in BLCA carcinogenesis, with increased *NCAM1* expression reported to enhance cell adhesion [47]. In contrast, *CBX7* exhibited a protective effect, with lower expression correlated with a reduced risk score in this study. *CBX7* is significantly downregulated in urinary bladder cancer (UBC) and correlates with poor prognosis. Overexpression of *CBX7* inhibits tumorigenicity, while its depletion promotes tumor growth. These results suggest a tumor-suppressive role for *CBX7* in UBC [48]. Therefore, the inclusion of key genes such as *MYC*, *ALDH1A1*, and *NCAM1* highlights their important roles in regulating cell cycle progression, maintaining stemness, and enabling immune evasion during BLCA pathogenesis [35,45,47,49].

Single-cell analysis showed that the prognostic characteristics of eight genes confirmed previous research results. DCN is restricted to fibroblasts [50,51], *NCAM1* to NK and endothelial cells [52], FASN to epithelium [53], and CSPG4 to fibroblast-rich subsets [54], whereas *ALDH1A1*, *CBX7*, *INHBB* and *MYC* [55] are sparsely expressed across lineages. Ligand–receptor inference identifies decorin (*DCN*) as the dominant stromal hub, bridging fibroblasts with myeloid (TLR2/4) and epithelial (EGFR/MET) cells and thereby coupling immune activation to epithelial proliferation. Additional niche-restricted signals include fibroblast-to-epithelial *CSPG4* → *ITGA3/ITGB1* [54], an endothelial autocrine *INHBB* → *SMAD3* loop, and low-intensity *NCAM1* contacts between endothelial and NK cells [52], while the remaining genes appear to act intracellularly. These findings position *DCN* and its cognate receptors as prime therapeutic targets [56] and nominate *CSPG4* for stromal-directed immunotherapies [57]. 

Our study also highlights the immunological differences between risk groups. Immune infiltration analysis showed that high-risk patients had increased levels of immunosuppressive cells, such as M2 macrophages and regulatory T cells (Tregs). This suggests that CSCs may contribute to an immunosuppressive tumor microenvironment (TME). Macrophage subtypes M0, M1, and M2 play distinct roles in the TME, influencing tumor growth, metastasis, and immune responses [58,59]. In this study, the high-risk group showed higher proportions of these macrophage subtypes, indicating a heightened inflammatory state and immune modulation within the TME. In the high-risk group, more M0 macrophages may indicate a greater abundance of unactivated monocytes. Additionally, chronic inflammation levels are elevated in the TME, which can influence the immune microenvironment via immune-related genes. *TNFRSF8* (*CD30*) and *CD44* are associated with modulating macrophage activity and promoting tumor migration and invasion, respectively [60,61]. Moreover, increased expression of immune checkpoint molecules, such as *PD-L1* (*CD274*) and *PD-L2* (*PDCD1LG2*), in high-risk patients suggests enhanced tumor immune evasion [62,63,64].

Conversely, genes such as *TNFSF14* (*LIGHT*) and *TNFRSF14* (*HVEM*), which promote anti-tumor immunity, showed higher expression in the low-risk group [35,36]. Additionally, lower *LAIR1* levels in this group enhance macrophage activity and inflammatory responses, thereby reducing tumor progression [65,66]. This immune landscape further underscores the intricate interplay between CSCs and immune modulation in BLCA. We propose this gene signature primarily as a robust prognostic and predictive biomarker, rather than as an immediate source of druggable targets. However, we observed differences in drug sensitivity between the risk groups. The low-risk group showed greater sensitivity to acetalax and afuresertib, possibly due to enhanced apoptotic pathways [55,56]. In contrast, the high-risk group’s sensitivity to paclitaxel and cisplatin may result from changes in microtubule dynamics or their function as anti-cancer immunomodulators [67,68]. These findings may help identify BLCA patients with distinct prognoses and treatment responses, allowing for more personalized therapies.

Despite the promising results, this study has some limitations. First, the sample size in the external validation cohorts was relatively small. This may limit the generalizability of our model. Larger, multi-center studies are needed to validate the prognostic power of the CSC gene signature in diverse populations. ScRNA-seq of BLCA revealed that *DCN* is the principal stromal communicator. *CSPG4*, *INHBB*, and *NCAM1* occupy specialized signaling niches. The data highlights the signature’s ability to capture the critical tumor-stroma-immune circuitry that could drive disease progression. However, validation in independent BLCA cohorts and functional perturbations are needed to confirm causality. Additionally, it remains unclear whether this signature can predict responses to specific treatments, such as chemotherapy or immunotherapy. Future studies should integrate treatment response data to assess the model’s predictive value in different therapeutic settings. Validation using prospective data or ITT cohorts should be performed to enhance confidence in the results. Although many reviews detail BCSCs, our study utilizes the most reliable BCSC gene sets from the dbBCSC database applied in pan-cancer research, rather than focusing solely on BCSCs specifically related to BLCA [69]. Another limitation is the reliance on bulk RNA sequencing data, which may obscure CSC heterogeneity within tumors.

Our BCSC-derived gene signature stratifies prognosis in BLCA. However, several future research directions could enhance the biological and clinical relevance of this signature. First, large prospective multicenter studies should validate the model across diverse cohorts. Second, integrating treatment response data, such as chemotherapy or immunotherapy outcomes, would help evaluate the signature’s predictive utility and support personalized treatment. Furthermore, scRNA-seq could reveal intratumoral heterogeneity and spatial gene expression within the tumor microenvironment. Additionally, functional experiments should be conducted to investigate key genes like *DCN*, *CSPG4*, and *INHBB* and clarify their causal roles. Finally, exploring these genes as therapeutic targets, especially in combination with immunotherapy, could advance precision medicine in BLCA.

## 5. Conclusions

We developed a CSC-based prognostic model that accurately predicts survival in BLCA patients. This model also elucidates the role of CSCs in the immune microenvironment and drug sensitivity. This model improves the understanding of BLCA pathogenesis and holds clinical potential for risk stratification and personalized therapy. Overall, these exploratory data serve as a valuable resource for generating hypotheses; however, these findings are limited to a specific dataset, so their generalizability requires further verification. Future research should validate the model in larger cohorts and explore its potential to guide immunotherapy strategies to improve BLCA outcomes.

## Data Availability

The datasets used in this study are publicly available from the following sources. The TCGA-BLCA dataset can be accessed through The Cancer Genome Atlas. The datasets GSE13507, GSE32894, and GSE222315 are accessible via the NCBI Gene Expression Omnibus. Additionally, Cancer Stem Cell Biomarkers (BCSCs) data are accessible from the BCSC Database and relevant references. TCGA-BLCA: https://portal.gdc.cancer.gov/ (accessed on 31 March 2024). GSE13507: https://www.ncbi.nlm.nih.gov/geo/query/acc.cgi?acc=GSE13507 (accessed on 31 March 2024). GSE32894: https://www.ncbi.nlm.nih.gov/geo/query/acc.cgi?acc=GSE32894 (accessed on 31 March 2024). GSE222315: https://www.ncbi.nlm.nih.gov/geo/query/acc.cgi?acc=GSE222315 (accessed on 31 March 2024). Cancer Stem Cell Biomarkers (BCSCs): http: //dibresources.jcbose.ac.in/ssaha4/bcscdb (accessed on 31 March 2024). All datasets are openly accessible and can be retrieved via the links provided for replication and further analysis.

## Supplementary Materials

The following supporting information can be downloaded at: https://www.mdpi.com/article/10.3390/genes16101146/s1. Figure S1: Flow diagram of this study illustrating the design and workflow. (Abbreviations: TCGA, The Cancer Genome Atlas; BLCA, Bladder Cancer; CSC, Cancer Stem Cell; DE-BCSC, Differentially Expressed Biomarkers of CSC; LASSO, Least Absolute Shrinkage and Selection Operator; DEGs, Differentially Expressed Genes; ICIs, Immune Checkpoint Inhibitors; GSEA, Gene Set Enrichment Analysis; GEO, Gene Expression Omnibus); Figure S2: Validation of Eight CSC Markers in BLCA tissue Samples. (A) The Volcano Plot illustrates differential expression of 120 CSC genes in TCGA-BLCA. (B) The expression of the eight genes between high-risk and low-risk groups within the GSE13507 cohort. (C) The ex-pression of the eight genes between high-risk and low-risk groups within the GSE32894 cohort; Figure S3: Pathway Enrichment Analysis of Differentially Expressed Genes in High- and Low-Risk Groups within the TCGA-BLCA Samples. (A) Analysis of the top 10 enriched biological processes (BP), cellular components (CC), and molecular functions (MF) based on the Gene Ontology (GO) database. (B) Top 30 Enriched Pathways in Kyoto Encyclopedia of Genes and Genomes (KEGG); Table S1: CSC-related genes; Table S2: Top enrichment functions obtained by GO and KEGG analysis based on prognostic risk scores; Table S3: Top enrichment functions obtained by GSEA based on prognostic risk scores; Table S4: The list of 120 differentially expressed Cancer Stem Cell (CSC) genes in bladder cancer (BLCA); Table S5: Timer 2.0 exploration modules explore associations between 8 signature gene expressions and tumor features in TCGA-BLCA.

## Abbreviations

The following abbreviations are used in this manuscript:

CSCs	Cancer stem cells
BCSCs	Biomarkers of CSCs
DE-BCSCs	Differentially expressed BCSCs
TCGA	The Cancer Genome Atlas
GEO	Gene Expression Omnibus
scRNA-seq	single-cell RNA sequencing
GO	Gene Ontology
KEGG	Kyoto Encyclopedia of Genes and Genomes
LASSO	Least Absolute Shrinkage and Selection Operator
OS	Overall Survival
TME	Tumor microenvironment
BLCA	Bladder cancer
EMT	Epithelial–Mesenchymal Transition
UMAP	Uniform Manifold Approximation and Projection
GSEA	Gene Set Enrichment Analysis
LIANA	Ligand–receptor ANalysis frAmework
