# Supplementary material for: Integrative Analysis of Biomarkers for Cancer Stem Cells in Bladder Cancer and Their Therapeutic Potential
Source: Genes (Basel). 2025 Sep 27;16(10):1146. doi: 10.3390/genes16101146 (PMC12563593; doi:10.3390/genes16101146)
Supplement: Supplementary file 1 [file genes-16-01146-s001.zip › Supplymentary_Table 2.pdf]

**S2 Table: Top enrichment function obtained by GO and KEGG analysis based on prognostic risk scores**

| Category | ID | Description | Count | p.adjust |
|---|---|---|---|---|
| **GO Biological Processes** | GO:0007159 | leukocyte cell-cell adhesion | 134 | 6.08E-23 |
| **GO Biological Processes** | GO:0022407 | regulation of cell-cell adhesion | 145 | 3.81E-21 |
| **GO Biological Processes** | GO:1903037 | regulation of leukocyte cell-cell adhesion | 122 | 3.81E-21 |
| **GO Biological Processes** | GO:0045785 | positive regulation of cell adhesion | 143 | 3.81E-21 |
| **GO Biological Processes** | GO:0050863 | regulation of T cell activation | 121 | 7.03E-21 |
| **GO Biological Processes** | GO:0022409 | positive regulation of cell-cell adhesion | 109 | 7.03E-21 |
| **GO Biological Processes** | GO:1903039 | positive regulation of leukocyte cell-cell adhesion | 98 | 1.11E-20 |
| **GO Biological Processes** | GO:0002696 | positive regulation of leukocyte activation | 120 | 1.55E-20 |
| **GO Biological Processes** | GO:0050867 | positive regulation of cell activation | 122 | 8.42E-20 |
| **GO Biological Processes** | GO:0050900 | leukocyte migration | 121 | 1.96E-19 |
| **GO Cellular Components** | GO:0062023 | collagen-containing extracellular matrix | 113 | 1.33E-13 |
| **GO Cellular Components** | GO:0030667 | secretory granule membrane | 79 | 3.73E-07 |
| **GO Cellular Components** | GO:0009897 | external side of plasma membrane | 92 | 1.32E-05 |
| **GO Cellular Components** | GO:0030666 | endocytic vesicle membrane | 52 | 1.32E-05 |
| **GO Cellular Components** | GO:0000940 | outer kinetochore | 10 | 3.13E-05 |
| **GO Cellular Components** | GO:0098636 | protein complex involved in cell adhesion | 22 | 7.13E-05 |
| **GO Cellular Components** | GO:0045178 | basal part of cell | 65 | 8.44E-05 |
| **GO Cellular Components** | GO:0000793 | condensed chromosome | 62 | 0.000102041 |
| **GO Cellular Components** | GO:0070820 | tertiary granule | 43 | 0.000102041 |
| **GO Cellular Components** | GO:0030669 | clathrin-coated endocytic vesicle membrane | 25 | 0.000102041 |
| **GO Molecular Functions** | GO:0140375 | immune receptor activity | 51 | 3.01E-09 |
| **GO Molecular Functions** | GO:0005201 | extracellular matrix structural constituent | 53 | 1.69E-07 |
| **GO Molecular Functions** | GO:0005125 | cytokine activity | 65 | 7.03E-07 |
| **GO Molecular Functions** | GO:0005178 | integrin binding | 46 | 5.70E-06 |
| **GO Molecular Functions** | GO:0038024 | cargo receptor activity | 31 | 5.94E-06 |
| **GO Molecular Functions** | GO:0032395 | MHC class II receptor activity | 9 | 3.01E-05 |
| **GO Molecular Functions** | GO:0008009 | chemokine activity | 21 | 5.94E-05 |
| **GO Molecular Functions** | GO:0005126 | cytokine receptor binding | 65 | 8.55E-05 |
| **GO Molecular Functions** | GO:0005539 | glycosaminoglycan binding | 58 | 8.55E-05 |
| **GO Molecular Functions** | GO:0072341 | modified amino acid binding | 28 | 0.000293187 |
| **KEGG Pathway** | hsa04640 | Hematopoietic cell lineage | 43 | 1.68E-11 |
| **KEGG Pathway** | hsa04061 | Viral protein interaction with cytokine and cytokine receptor | 43 | 1.68E-11 |
| **KEGG Pathway** | hsa04060 | Cytokine-cytokine receptor interaction | 85 | 4.71E-11 |
| **KEGG Pathway** | hsa05323 | Rheumatoid arthritis | 40 | 6.10E-11 |
| **KEGG Pathway** | hsa04514 | Cell adhesion molecules | 55 | 9.83E-11 |
| **KEGG Pathway** | hsa05140 | Leishmaniasis | 35 | 1.59E-10 |
| **KEGG Pathway** | hsa04110 | Cell cycle | 50 | 2.77E-08 |
| **KEGG Pathway** | hsa04145 | Phagosome | 47 | 2.34E-07 |
| **KEGG Pathway** | hsa05150 | Staphylococcus aureus infection | 34 | 6.35E-07 |
| **KEGG Pathway** | hsa04672 | Intestinal immune network for IgA production | 22 | 1.44E-06 |
