# Supplementary material for: Integrative Analysis of Biomarkers for Cancer Stem Cells in Bladder Cancer and Their Therapeutic Potential
Source: Genes (Basel). 2025 Sep 27;16(10):1146. doi: 10.3390/genes16101146 (PMC12563593; doi:10.3390/genes16101146)
Supplement: Supplementary file 1 [file genes-16-01146-s001.zip › Supplymentary_Table 3.pdf]

S3 Table: Top enrichment function obtained by GSEA analysis based on prognostic risk scores

Category	Description	EnrichmentScore	NES	Pvalue
**KEGG Pathway**	KEGG_ECM_RECEPTOR_INTERACTION	0.780993	2.427339	1.00E-10
**KEGG Pathway**	KEGG_FOCAL_ADHESION	0.674636	2.331778	1.00E-10
**KEGG Pathway**	KEGG_COMPLEMENT_AND_COAGULATION_CASCADES	0.737002	2.237102	1.00E-10
**KEGG Pathway**	KEGG_ARRHYTHMOGENIC_RIGHT_VENTRICULAR_CARDIOMYOPATHY_ARVC	0.668202	2.065691	1.25E-07
**KEGG Pathway**	KEGG_SYSTEMIC_LUPUS_ERYTHEMATOSUS	0.707326	2.007435	8.03E-06
**KEGG Pathway**	KEGG_PRION_DISEASES	0.74047	1.990904	2.07E-05
**KEGG Pathway**	KEGG_CYTOKINE_CYTOKINE_RECEPTOR_INTERACTION	0.557026	1.985865	1.00E-10
**KEGG Pathway**	KEGG_DILATED_CARDIOMYOPATHY	0.628942	1.977482	2.64E-07
**KEGG Pathway**	KEGG_HYPERTROPHIC_CARDIOMYOPATHY_HCM	0.634156	1.976704	3.68E-07
**KEGG Pathway**	KEGG_HEMATOPOIETIC_CELL_LINEAGE	0.62778	1.954649	6.74E-07
**KEGG Pathway**	KEGG_REGULATION_OF_ACTIN_CYTOSKELETON	0.545979	1.910152	7.36E-09
**KEGG Pathway**	KEGG_CELL_CYCLE	0.577238	1.896093	1.22E-06
**KEGG Pathway**	KEGG_LEISHMANIA_INFECTION	0.631236	1.866028	5.42E-05
**KEGG Pathway**	KEGG_CHEMOKINE_SIGNALING_PATHWAY	0.537613	1.85797	7.57E-08
**KEGG Pathway**	KEGG_PATHOGENIC_ESCHERICHIA_COLI_INFECTION	0.630999	1.85509	0.00015
**KEGG Pathway**	KEGG_GAP_JUNCTION	0.586719	1.837413	2.18E-05
**KEGG Pathway**	KEGG_GRAFT_VERSUS_HOST_DISEASE	0.757079	1.819302	0.000439
**KEGG Pathway**	KEGG_GLYCOSAMINOGLYCAN_BIOSYNTHESIS_CHONDROITIN_SULFATE	0.691472	1.713295	0.006032
**KEGG Pathway**	KEGG_VASCULAR_SMOOTH_MUSCLE_CONTRACTION	0.524142	1.697583	0.000119
**KEGG Pathway**	KEGG_VIRAL_MYOCARDITIS	0.575303	1.657318	0.004959
**KEGG Pathway**	KEGG_RENAL_CELL_CARCINOMA	0.547702	1.656142	0.002344
**KEGG Pathway**	KEGG_TOLL_LIKE_RECEPTOR_SIGNALING_PATHWAY	0.516051	1.652828	0.000376
**KEGG Pathway**	KEGG_CELL_ADHESION_MOLECULES_CAMS	0.509206	1.646174	0.00032
**KEGG Pathway**	KEGG_MELANOMA	0.532893	1.626011	0.002286
**KEGG Pathway**	KEGG_GLIOMA	0.536891	1.619647	0.002317
**KEGG Pathway**	KEGG_NOD_LIKE_RECEPTOR_SIGNALING_PATHWAY	0.541916	1.61698	0.002834
**KEGG Pathway**	KEGG_DNA_REPLICATION	0.592929	1.611377	0.007046
**KEGG Pathway**	KEGG_OOCYTE_MEIOSIS	0.492863	1.596275	0.000964

**KEGG Pathway**	KEGG_PATHWAYS_IN_CANCER	0.440408	1.595093	1.61E-05
**KEGG Pathway**	KEGG_SMALL_CELL_LUNG_CANCER	0.506802	1.579734	0.002996
**KEGG Pathway**	KEGG_LEUKOCYTE_TRANSENDOTHELIAL_MIGRATION	0.483841	1.579285	0.002757
**KEGG Pathway**	KEGG_PROGESTERONE_MEDIATED_OOCYTE_MATURATION	0.491527	1.534984	0.004413
**KEGG Pathway**	KEGG_COLORECTAL_CANCER	0.506279	1.510645	0.010231
**KEGG Pathway**	KEGG_WNT_SIGNALING_PATHWAY	0.437702	1.478965	0.009736
**KEGG Pathway**	KEGG_JAK_STAT_SIGNALING_PATHWAY	0.432982	1.466431	0.009123
**KEGG Pathway**	KEGG_ADHERENS_JUNCTION	0.483269	1.465761	0.013227
**KEGG Pathway**	KEGG_MELANOGENESIS	0.454349	1.451322	0.011353
**KEGG Pathway**	KEGG_NATURAL_KILLER_CELL_MEDIATED_CYTOTOXICITY	0.436108	1.436906	0.008581
**KEGG Pathway**	KEGG_CALCIUM_SIGNALING_PATHWAY	0.406136	1.395227	0.01156
**KEGG Pathway**	KEGG_MAPK_SIGNALING_PATHWAY	0.390253	1.389388	0.004285
**KEGG Pathway**	KEGG_ARACHIDONIC_ACID_METABOLISM	-0.45962	-1.50517	0.011288
**KEGG Pathway**	KEGG_TASTE_TRANSDUCTION	-0.46513	-1.51152	0.011725
**KEGG Pathway**	KEGG_GLYCEROPHOSPHOLIPID_METABOLISM	-0.44834	-1.52083	0.006458
**KEGG Pathway**	KEGG_OXIDATIVE_PHOSPHORYLATION	-0.43719	-1.58773	0.001113
**KEGG Pathway**	KEGG_PORPHYRIN_AND_CHLOROPHYLL_METABOLISM	-0.51522	-1.59199	0.010514
**KEGG Pathway**	KEGG_PENTOSE_AND_GLUCURONATE_INTERCONVERSIONS	-0.57083	-1.6117	0.012238
**KEGG Pathway**	KEGG_LINOLEIC_ACID_METABOLISM	-0.6005	-1.69547	0.005586
**KEGG Pathway**	KEGG_PEROXISOME	-0.5075	-1.74481	0.000474
**KEGG Pathway**	KEGG_STEROID_HORMONE_BIOSYNTHESIS	-0.56712	-1.83599	0.000451
**KEGG Pathway**	KEGG_RETINOL_METABOLISM	-0.55208	-1.83666	0.000243
**KEGG Pathway**	KEGG_DRUG_METABOLISM_CYTOCHROME_P450	-0.59535	-2.01949	2.78E-06
**KEGG Pathway**	KEGG_METABOLISM_OF_XENOBIOTICS_BY_CYTOCHROME_P450	-0.6656	-2.25374	1.79E-08
**KEGG Pathway**	KEGG_RIBOSOME	-0.71172	-2.52858	1.00E-10
**HALLMARK**	HALLMARK_EPITHELIAL_MESENCHYMAL_TRANSITION	0.848504	2.94307	1.00E-10
**HALLMARK**	HALLMARK_TNFA_SIGNALING_VIA_NFKB	0.677173	2.348802	1.00E-10
**HALLMARK**	HALLMARK_G2M_CHECKPOINT	0.675057	2.333708	1.00E-10
**HALLMARK**	HALLMARK_E2F_TARGETS	0.641144	2.225663	1.00E-10
**HALLMARK**	HALLMARK_INFLAMMATORY_RESPONSE	0.634279	2.197447	1.00E-10

**HALLMARK**	HALLMARK_COAGULATION	0.651009	2.164076	1.00E-10
**HALLMARK**	HALLMARK_APICAL_JUNCTION	0.616419	2.138304	1.00E-10
**HALLMARK**	HALLMARK_MYOGENESIS	0.6135	2.127949	1.00E-10
**HALLMARK**	HALLMARK_MTORC1_SIGNALING	0.612944	2.12777	1.00E-10
**HALLMARK**	HALLMARK_ANGIOGENESIS	0.784455	2.126917	1.97E-07
**HALLMARK**	HALLMARK_HYPOXIA	0.604867	2.0953	1.00E-10
**HALLMARK**	HALLMARK_COMPLEMENT	0.582148	2.021352	1.00E-10
**HALLMARK**	HALLMARK_IL6_JAK_STAT3_SIGNALING	0.63335	1.991459	2.90E-07
**HALLMARK**	HALLMARK_UV_RESPONSE_DN	0.596557	1.978386	1.29E-08
**HALLMARK**	HALLMARK_KRAS_SIGNALING_UP	0.560903	1.947116	4.04E-09
**HALLMARK**	HALLMARK_ALLOGRAFT_REJECTION	0.562601	1.940499	8.40E-09
**HALLMARK**	HALLMARK_MITOTIC_SPINDLE	0.555892	1.927502	4.31E-09
**HALLMARK**	HALLMARK_APOPTOSIS	0.526382	1.778554	6.09E-06
**HALLMARK**	HALLMARK_IL2_STAT5_SIGNALING	0.511406	1.773849	1.12E-06
**HALLMARK**	HALLMARK_MYC_TARGETS_V1	0.503863	1.747859	5.47E-06
**HALLMARK**	HALLMARK_INTERFERON_GAMMA_RESPONSE	0.502484	1.740639	7.66E-06
**HALLMARK**	HALLMARK_UNFOLDED_PROTEIN_RESPONSE	0.536074	1.733778	0.00016
**HALLMARK**	HALLMARK_HEDGEHOG_SIGNALING	0.599114	1.604493	0.008815
**HALLMARK**	HALLMARK_GLYCOLYSIS	0.442503	1.534838	0.000493
**HALLMARK**	HALLMARK_TGF_BETA_SIGNALING	0.51215	1.499386	0.02123
**HALLMARK**	HALLMARK_CHOLESTEROL_HOMEOSTASIS	0.471041	1.437204	0.020863
**HALLMARK**	HALLMARK_SPERMATOGENESIS	0.411997	1.365306	0.023428
**HALLMARK**	HALLMARK_OXIDATIVE_PHOSPHORYLATION	-0.3321	-1.30379	0.024402
**HALLMARK**	HALLMARK_PEROXISOME	-0.38506	-1.39685	0.02121
