# Supplementary material for: Integrative Analysis of Biomarkers for Cancer Stem Cells in Bladder Cancer and Their Therapeutic Potential
Source: Genes (Basel). 2025 Sep 27;16(10):1146. doi: 10.3390/genes16101146 (PMC12563593; doi:10.3390/genes16101146)
Supplement: Supplementary file 1 [file genes-16-01146-s001.zip › Supplymentary_Table 4.pdf]

S4 Table: The list of 120 differentially expressed Cancer Stem Cell (CSC) genes in bladder cancer (BLCA).

gene_name	logFC	Average Expression	t-statistic	P.Value	Adjusted P-Value	B statistic
**ACTA2**	-3.368013969	5.768982588	-8.230317241	2.38E-15	6.77E-14	24.11748145
**AKAP12**	-2.202978739	2.154771686	-7.911498172	2.28E-14	5.55E-13	21.90014371
**ALDH1A1**	-1.704234814	2.512997328	-3.956855312	8.91E-05	0.000397377	0.503971388
**ALDH1B1**	-1.610486741	3.868872724	-6.553568189	1.65E-10	2.18E-09	13.21233702
**ANTXR2**	-1.805603163	2.260368677	-7.990530628	1.31E-14	3.34E-13	22.44382718
**APBB1**	-1.550799831	1.562085581	-7.333493621	1.16E-12	2.15E-11	18.04767478
**B3GNT3**	1.322602477	4.506989278	3.39898172	0.000741008	0.002659923	-1.475860682
**BCL2**	-1.057290376	1.074645149	-7.522400616	3.30E-13	6.65E-12	19.28240272
**BIRC5**	2.421955401	3.950890424	10.14612357	8.83E-22	6.19E-20	38.68203964
**BOP1**	1.296933445	4.632559154	7.867951024	3.09E-14	7.31E-13	21.60227825
**CACNA2D1**	-1.178342705	0.83053652	-6.559421056	1.59E-10	2.11E-09	13.2469998
**CAV1**	-1.752501331	4.507969562	-4.476173052	9.80E-06	5.30E-05	2.596264877
**CBX7**	-1.924942061	1.980937586	-11.67043241	1.89E-27	2.61E-25	51.55928101
**CCL2**	-2.24960802	3.005312512	-6.442194583	3.23E-10	4.00E-09	12.55755337
**CCNA2**	2.01545751	3.541250106	9.168668814	2.16E-18	9.13E-17	30.99902308
**CCNB1**	2.323261879	4.584330383	10.13065273	1.00E-21	6.90E-20	38.55667682
**CD24**	1.648288911	6.744199482	4.002171832	7.42E-05	0.000335883	0.677084278
**CD34**	-1.708677881	2.271657793	-9.297614026	7.94E-19	3.59E-17	31.98386634
**CDH1**	1.765790706	5.937263391	4.888551305	1.45E-06	9.28E-06	4.423949145
**CDK1**	2.392924507	3.744228939	11.80131102	5.91E-28	8.71E-26	52.70940848
**CDKN1A**	-1.129345826	6.079257693	-4.31146411	2.02E-05	0.000102549	1.907087839
**CHEK1**	1.130142834	2.118407825	7.565868588	2.46E-13	5.05E-12	19.56990525

**CLU**	-2.567923464	4.950504371	-5.558149352	4.85E-08	4.09E-07	7.694141071
**COL6A1**	-1.385518146	5.743863776	-3.528860743	0.000463465	0.001759344	-1.040136033
**COL7A1**	1.796853385	3.705535837	4.825954457	1.95E-06	1.22E-05	4.137218086
**CSF3**	-2.245720059	0.992010893	-6.748590614	4.97E-11	7.12E-10	14.38079668
**CSPG4**	-1.309497298	2.221248634	-4.815920314	2.05E-06	1.27E-05	4.091562842
**CTSG**	-2.221457234	0.919396887	-8.667136164	9.72E-17	3.40E-15	27.25691226
**CXCL2**	-1.897296898	1.840174608	-5.531922981	5.58E-08	4.67E-07	7.559188789
**CXCL9**	1.191580692	2.779780586	2.352129322	0.019127211	0.045666708	-4.415094929
**CXCR4**	-1.081921058	3.77303509	-3.618445529	0.000332493	0.001304643	-0.730627021
**CYR61**	-3.108139169	5.442936947	-8.535542359	2.58E-16	8.36E-15	26.29909955
**DCN**	-3.02188909	3.590658844	-7.398396555	7.57E-13	1.44E-11	18.46917555
**DDR1**	1.027262684	5.168734192	4.527484645	7.79E-06	4.31E-05	2.81576093
**DHCR7**	1.523120675	4.113117765	7.616221586	1.75E-13	3.68E-12	19.90451535
**DLGAP5**	1.806844008	2.77183109	8.262688625	1.88E-15	5.44E-14	24.34616723
**DUSP9**	1.001268931	1.532430445	3.338236814	0.000918298	0.003221488	-1.674345913
**E2F1**	1.866065292	3.549430691	7.343614416	1.09E-12	2.02E-11	18.11321427
**EGR1**	-3.309470638	5.461432005	-9.452044588	2.37E-19	1.19E-17	33.17519256
**EME1**	1.103784391	1.395527761	7.818729795	4.35E-14	1.01E-12	21.26707026
**EPCAM**	2.163632324	5.33563613	5.93162904	6.27E-09	6.24E-08	9.675088792
**ESM1**	1.881967873	2.060602853	6.68265442	7.48E-11	1.04E-09	13.98265077
**ETV4**	1.923331127	3.004158645	6.530088612	1.90E-10	2.47E-09	13.07353561
**EXO1**	1.33062911	1.868694803	7.709885905	9.21E-14	2.03E-12	20.53139234
**EZH2**	1.523505588	2.777338764	8.083952685	6.76E-15	1.82E-13	23.09160791
**FASN**	1.365803272	5.265052504	6.055458934	3.11E-09	3.28E-08	10.35594065
**FAXDC2**	-1.582834632	0.879695014	-12.2180515	1.39E-29	2.63E-27	56.41335286
**FGFR1**	-1.971528453	1.752809387	-7.785151376	5.48E-14	1.25E-12	21.03929177

**FOS**	-3.204496167	6.283264344	-8.742589966	5.53E-17	1.96E-15	27.81069639
**FOXF1**	-2.754860789	1.879678551	-11.39106281	2.23E-26	2.69E-24	49.12622072
**FOXM1**	2.168568558	3.408498711	8.599534874	1.61E-16	5.41E-15	26.76359436
**FSCN1**	1.43795717	5.800915199	4.27830872	2.33E-05	0.000116749	1.771212832
**FSTL1**	-1.007224464	4.546034792	-3.646777349	0.000298914	0.00118555	-0.631225529
**FXYD3**	1.733693102	6.853171719	3.767068048	0.000188768	0.000784652	-0.201103373
**FZD2**	1.315077237	2.385018995	5.689545939	2.39E-08	2.14E-07	8.378523265
**FZD7**	-1.681337855	2.633001208	-7.749288381	7.02E-14	1.58E-12	20.79682408
**GALNT3**	1.062677887	3.221001649	4.240734725	2.74E-05	0.000135007	1.618391634
**GATA6**	-1.99637588	1.185549982	-10.54365969	3.24E-23	2.55E-21	41.94219198
**GJB2**	1.845688047	5.146910927	3.531162683	0.000459567	0.001746587	-1.032274414
**GPC3**	-1.05820923	1.838739918	-2.943623039	0.003423983	0.010227823	-2.880672928
**GPT2**	1.110492091	2.873990035	5.165024921	3.72E-07	2.66E-06	5.729552118
**HMGA1**	1.712502354	6.996452736	7.198084893	2.84E-12	4.91E-11	17.17754172
**IGFBP3**	1.179476535	6.962517015	2.673834221	0.0077915	0.020846572	-3.621646526
**IL33**	-2.109868483	1.841633069	-6.117104011	2.18E-09	2.36E-08	10.69927982
**IL6**	-2.877396578	1.634643598	-8.185318775	3.28E-15	9.23E-14	23.80066755
**INHBA**	1.195797835	1.978613018	3.728484893	0.000219048	0.000897406	-0.340487715
**INHBB**	-1.00981936	1.715154132	-4.508484375	8.48E-06	4.65E-05	2.734218691
**ITGB4**	1.157117769	5.991980033	3.481025872	0.000551837	0.002052783	-1.202408606
**JUN**	-1.876621118	5.783625159	-6.819879545	3.19E-11	4.74E-10	14.8147919

**JUNB**	-1.403333137	7.496849991	-6.406053482	4.01E-10	4.88E-09	12.34704765
**JUP**	1.442320153	7.3185545	5.44335215	8.91E-08	7.17E-07	7.107512973
**KCNE4**	-1.528427804	1.155594909	-7.35535298	1.01E-12	1.88E-11	18.18931724
**KCNK3**	-1.262924922	0.499816374	-8.054604455	8.33E-15	2.22E-13	22.88751676
**KIF11**	1.683816517	3.054432005	8.571675456	1.97E-16	6.56E-15	26.56107595
**KIT**	-1.224143327	1.289009898	-4.470182697	1.01E-05	5.43E-05	2.570787957
**KLF4**	-2.313845144	3.375400102	-8.598998261	1.61E-16	5.42E-15	26.75968922
**KRT19**	2.090831962	9.548512605	4.200081922	3.26E-05	0.000158023	1.45444167
**LIG1**	1.345061046	3.348779605	9.781798031	1.71E-20	9.87E-19	35.76103864
**LRIG1**	-1.04056789	1.653859137	-4.055222751	5.97E-05	0.000276204	0.882061401
**MAGEA3**	1.529859075	1.884988572	2.918530725	0.003705937	0.010945782	-2.952475201
**MAOB**	-2.782449208	1.506081085	-9.365679336	4.67E-19	2.19E-17	32.50736771
**MKI67**	2.041678518	3.198164533	9.369241592	4.54E-19	2.14E-17	32.53483425
**MMP9**	1.30628367	3.035763049	2.785812384	0.005581249	0.015698774	-3.322421101
**MYC**	-1.531252506	4.630399813	-4.539392881	7.38E-06	4.10E-05	2.867025167
**NCAM1**	-2.172518385	0.565234295	-11.19913128	1.19E-25	1.30E-23	47.4725944
**NES**	-1.30857445	2.824274063	-4.911167635	1.30E-06	8.41E-06	4.528355904
**NGFR**	-1.172378288	1.180396403	-4.182799848	3.51E-05	0.000168918	1.385183786
**NR4A2**	-2.484955415	2.152493065	-10.64523767	1.37E-23	1.16E-21	42.78692112
**ODC1**	-1.357136658	4.57821841	-4.921691344	1.23E-06	8.03E-06	4.577084224
**OLFM4**	-1.425924628	1.609557826	-2.554628754	0.010981953	0.028115533	-3.927140368
**PCNA**	1.560305091	6.636151521	7.978515845	1.42E-14	3.61E-13	22.36091829
**PLAU**	1.702352689	5.32298292	4.481307317	9.58E-06	5.19E-05	2.618125521
**PRR11**	1.682016956	2.718604418	8.042833806	9.05E-15	2.39E-13	22.80581456

**PTGS2**	-2.326580036	2.990914507	-5.947332438	5.74E-09	5.74E-08	9.760774402
**PTK7**	1.028643841	3.78682548	4.571623732	6.38E-06	3.59E-05	3.006388578
**RAD51**	1.531745258	2.460375405	9.442392806	2.55E-19	1.27E-17	33.1003623
**RHOB**	-2.040693302	6.408665963	-9.110575867	3.38E-18	1.40E-16	30.55830926
**RND3**	-1.262806895	4.207668952	-5.022298071	7.56E-07	5.11E-06	5.047603906
**S100B**	-1.132275067	1.467652318	-4.580357214	6.13E-06	3.46E-05	3.044305006
**SCD**	1.781314357	5.674270148	5.249636244	2.43E-07	1.80E-06	6.141759645
**SCUBE2**	-1.168364767	2.311054847	-2.686198469	0.007513711	0.020256433	-3.589189074
**SFN**	2.150754353	8.390521002	4.977772763	9.41E-07	6.25E-06	4.838324159
**SHH**	-1.851912679	1.395490224	-4.591460326	5.82E-06	3.30E-05	3.092603654
**SKP2**	1.542937366	3.524219793	7.767613344	6.19E-14	1.40E-12	20.92061364
**SLC2A3**	-1.58215472	2.550218362	-5.328308272	1.62E-07	1.24E-06	6.530291888
**SMUG1**	1.021426472	3.124327023	9.040618084	5.79E-18	2.33E-16	30.03007027
**SOX4**	1.705120555	4.970926259	6.703827075	6.56E-11	9.18E-10	14.11015569
**SOX9**	1.050721042	2.7854646	2.851330656	0.004568455	0.013140468	-3.141861334
**TACSTD2**	1.90478504	8.085843686	4.685725015	3.77E-06	2.22E-05	3.506905288
**TGFB3**	-1.307631791	2.091011707	-5.062654017	6.20E-07	4.25E-06	5.238712465
**TGFBR2**	-1.330410293	3.996119326	-6.158081552	1.72E-09	1.90E-08	10.92911564
**THRA**	-1.189864944	2.237412207	-7.339167085	1.12E-12	2.08E-11	18.08440599
**TIMP2**	-1.523513394	4.721617769	-4.149920371	4.03E-05	0.000191668	1.254144657
**TUBB**	1.009875988	8.131801205	6.609909318	1.17E-10	1.58E-09	13.54705228
**TWIST2**	-1.558178987	0.977923477	-7.326289469	1.22E-12	2.25E-11	18.00106515
**UNG**	1.113006683	4.673981723	8.247624816	2.10E-15	6.03E-14	24.2396695
**VCL**	-1.209209271	4.141915496	-6.639726708	9.74E-11	1.33E-09	13.72513294
**VIM**	-1.355828756	5.945234478	-4.148400015	4.05E-05	0.0001928	1.248108395

**ZEB1**	-1.746106828	1.450097048	-9.08631231	4.08E-18	1.67E-16	30.37479066
**ZEB2**	-1.115094392	0.88774964	-8.312046801	1.32E-15	3.86E-14	24.69609493
