# Supplementary figures and images for: Integrative Analysis of Biomarkers for Cancer Stem Cells in Bladder Cancer and Their Therapeutic Potential
Source: Genes (Basel). 2025 Sep 27;16(10):1146. doi: 10.3390/genes16101146 (PMC12563593; doi:10.3390/genes16101146)

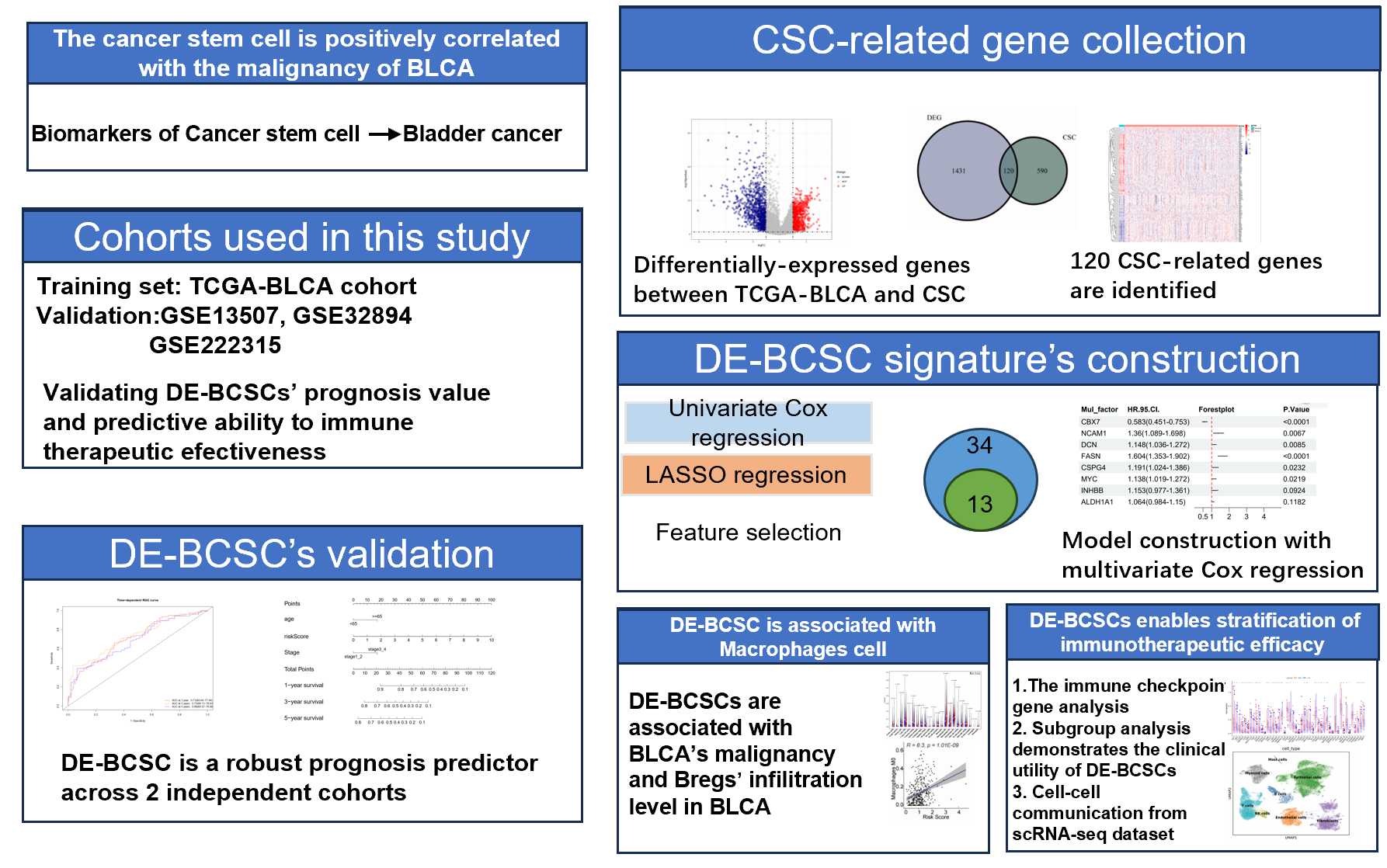

Supplement: Supplementary file 1 [file genes-16-01146-s001.zip › Figure S1.png]

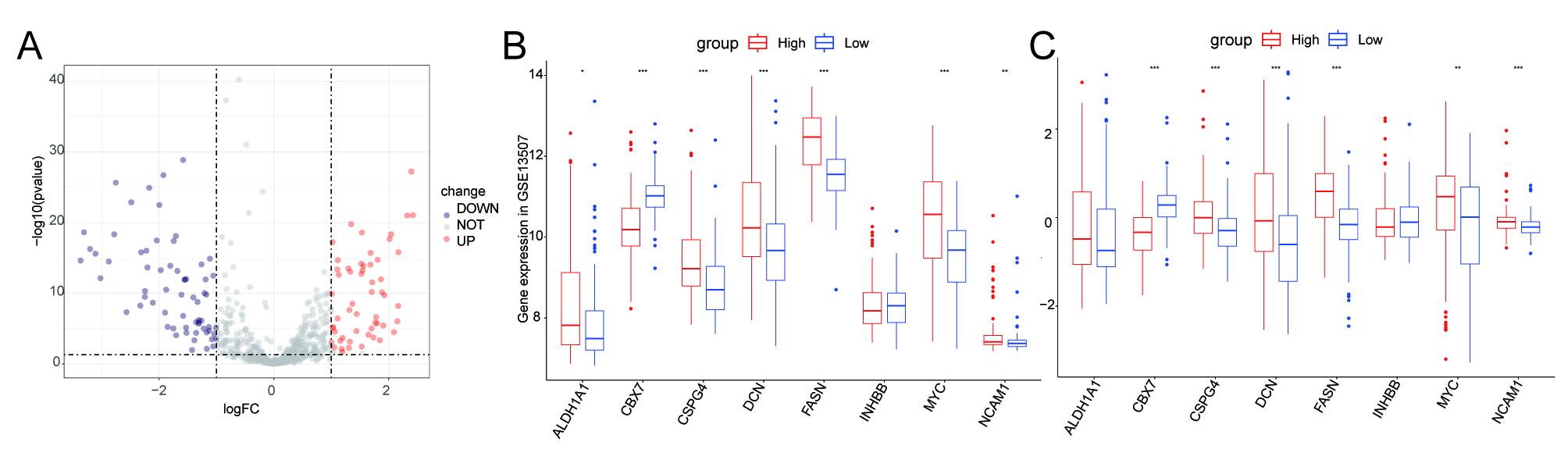

Supplement: Supplementary file 1 [file genes-16-01146-s001.zip › FigureS2.tif]

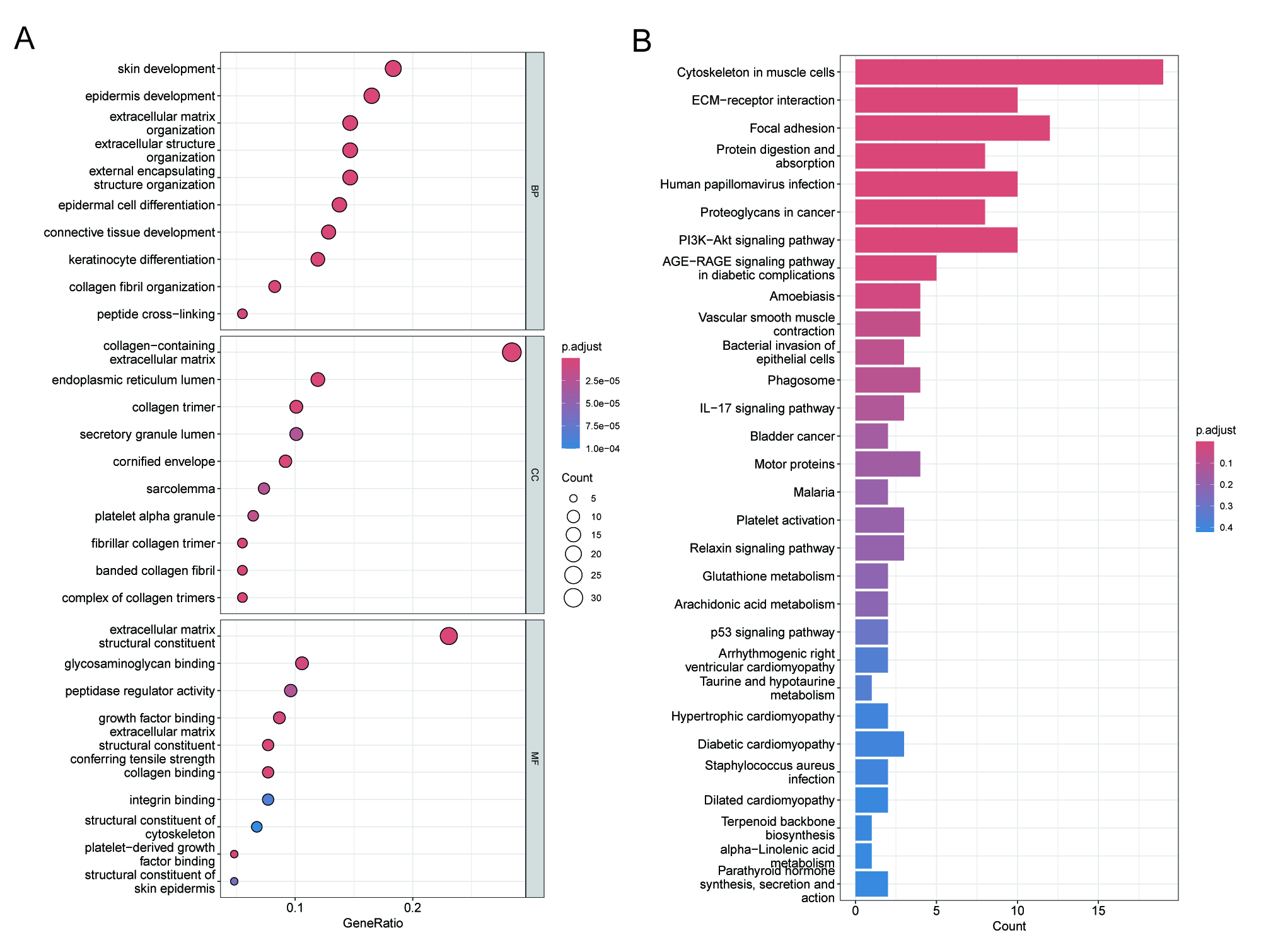

Supplement: Supplementary file 1 [file genes-16-01146-s001.zip › FigureS3.tif]
